# Carcinogenesis of Pancreatic Adenocarcinoma: Precursor Lesions

**DOI:** 10.3390/ijms141019731

**Published:** 2013-09-30

**Authors:** Antonio Gnoni, Antonella Licchetta, Aldo Scarpa, Amalia Azzariti, Anna Elisabetta Brunetti, Gianni Simone, Patrizia Nardulli, Daniele Santini, Michele Aieta, Sabina Delcuratolo, Nicola Silvestris

**Affiliations:** 1 Medical Oncology Unit, Hospital Vito Fazzi, Lecce 73100, Italy; E-Mails: drgnoni.antonio@libero.it (A.G.); antonellalicchetta@libero.it (A.L.); 2 Department of Pathology and Diagnostics, University of Verona, Verona 37121, Italy; E-Mail: aldo.scarpa@univr.it; 3 Clinical and Preclinical Pharmacology Laboratory, National Cancer Research Centre Istituto Tumori “Giovanni Paolo II”, Bari 70124, Italy; E-Mail: a.azzariti@oncologico.bari.it; 4 Scientific Direction, National Cancer Research Centre Istituto Tumori “Giovanni Paolo II”, Bari 70124, Italy; E-Mail: brunetti.ae@gmail.com (A.E.B.); sabinadelcuratolo@libero.it (S.D.); 5 Histopathology Unit, National Cancer Research Centre Istituto Tumori “Giovanni Paolo II”, Bari 70124, Italy; E-Mail: g.simone@oncologico.bari.it; 6 Hospital Pharmacy Unit - National Cancer Research Centre Istituto Tumori “Giovanni Paolo II”, Bari 70124, Italy; E-Mail: p.nardulli@oncologico.bari.it; 7 Medical Oncology Department, University Campus Bio-Medico, Rome 00199, Italy; E-Mail: d.santini@unicampus.it; 8 Medical Oncology Unit - CROB-IRCCS, 85028, Rionero in Vulture, Potenza 85100, Italy; E-Mail: michele.aieta@crob.it; 9 Medical Oncology Unit, National Cancer Research Centre Istituto Tumori “Giovanni Paolo II”, Viale Orazio Flacco 65, Bari 70124, Italy

**Keywords:** carcinogenesis, micro-RNAs, oncogenes, pancreatic adenocarcinoma, precursor lesions

## Abstract

Pancreatic adenocarcinoma displays a variety of molecular changes that evolve exponentially with time and lead cancer cells not only to survive, but also to invade the surrounding tissues and metastasise to distant sites. These changes include: genetic alterations in oncogenes and cancer suppressor genes; changes in the cell cycle and pathways leading to apoptosis; and also changes in epithelial to mesenchymal transition. The most common alterations involve the *epidermal growth factor receptor* (*EGFR*) gene, the *HER2* gene, and the *K-ras* gene. In particular, the loss of function of tumor-suppressor genes has been documented in this tumor, especially in *CDKN2a*, *p53*, *DPC4* and *BRCA2* genes. However, other molecular events involved in pancreatic adenocarcinoma pathogenesis contribute to its development and maintenance, specifically epigenetic events. In fact, key tumor suppressors that are well established to play a role in pancreatic adenocarcinoma may be altered through hypermethylation, and oncogenes can be upregulated secondary to permissive histone modifications. Indeed, factors involved in tumor invasiveness can be aberrantly expressed through dysregulated microRNAs. This review summarizes current knowledge of pancreatic carcinogenesis from its initiation within a normal cell until the time that it has disseminated to distant organs. In this scenario, highlighting these molecular alterations could provide new clinical tools for early diagnosis and new effective therapies for this malignancy.

## 1. Introduction

Pancreatic ductal adenocarcinoma (PDAC) is an aggressive malignant disease of the exocrine pancreas with a 5-year survival of less than 5% [1]. It represents the fourth-leading cause of cancer-related deaths worldwide [2]. Men and women have an approximately equal risk and pancreatic cancer causes an estimated 213,000 deaths each year [3]. The majority of patients present with locally advanced or metastatic disease, with only about 15% of patients being candidates for surgical resection. In this small setting, European Study of Pancreatic and Ampullary Cancer (ESPAC) trials have consistently demonstrated a modest survival benefit associated with post-operative adjuvant therapy, with fluorouracil and gemcitabine proving equally effective [4].

Despite similar presentation and histological appearance, variants of PDAC, such as adenosquamous carcinoma, colloid carcinoma, *etc*. are biologically diverse and exhibit complex molecular and cellular heterogeneity. However, aggressive progression is common to all [5]. PDAC is rare before the age of 40, and the median age at diagnosis is 73 years. Long-term cigarette smoking represents the leading preventable cause, but other risk factors have been validated, including diets high in meats and fat, low serum folate levels, obesity, long-standing diabetes mellitus, and chronic pancreatitis [6]. Approximately 10% of patients demonstrate a familial predisposition for PDAC, and a subset of these patients harbor germline mutations in *BRCA2*, *P16/CDKN2A*, *PRSS1*, *STK11/LKB1*, or, rarely, in DNA mismatch repair genes. In the vast majority of patients with familial risk the underlying genetic predisposition remains unknown. After this evidence, algorithms and strategies for PDAC should change, taking into account also tumor biology [6].

Research over the last two decades has shown that PDAC is caused by inherited germline and acquired somatic mutations in cancer-associated genes, including oncogenes (*i.e.*, *KRAS*), tumor suppressor genes, cell cycle genes, apoptosis and genome-maintenance genes, determinant in pancreatic cancer progression and metastases. The dependence of PDAC on these factors and certain crucial inflammatory mediators suggests that targeting these central roles may hold hope for effective therapies. Many of these changes already appear in PDAC precursor lesions, which are discussed below.

## 2. Precursor Lesions

**Figure 1:**
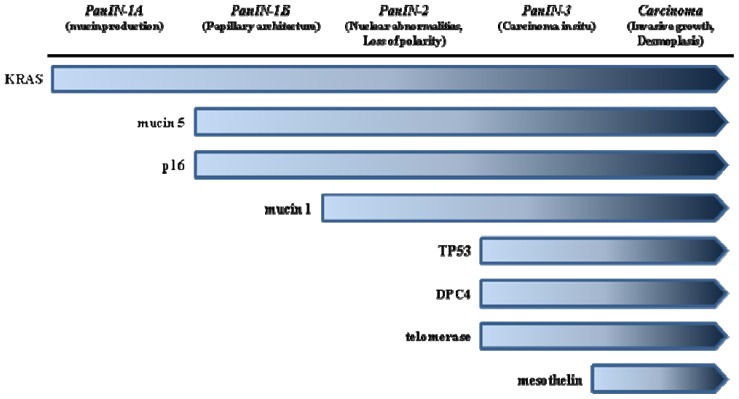
Precursor lesions in PDAC: correlation with oncogenes and tumor suppressor genes.

Three types of PDAC precursor lesions have been characterized in the last decade: pancreatic intraepithelial neoplasia (PanIN), intraductal papillary neoplasia (IPMNs) and mucinous cystic neoplasms (MCNs). They all follow a multistep progression to invasive cancer characterized by increasing degrees of morphological and cytological atypia [7] (Figure 1). The following observations are in favor of a multistep carcinogenesis: duct lesions are far more common in pancreases with infiltrating carcinoma [8]; there is an increase in the grade of lesions surrounding infiltrating carcinoma [9]; patients diagnosed with intraductal mucinous neoplasms that are not resected subsequently develop infiltrating carcinoma [9]. All three known precursor lesions bear ductal epithelial cell characteristics, but the precise cellular origin of these lesions is still highly debated. The contribution of immature pancreatic precursors to PDAC progression raises the possibility of the presence of pancreatic cancer stem cells (CSC). These cells give rise to a heterogeneous lineage [10,11]. Indeed, CSC may derive from this particular population, to acquire a slightly transformed phenotype, known as “minimal deviation” [12]. The location and the rarity of these cells within the tumor would contribute to explain their resistance against conventional therapies, and there is a correlation with the “epithelial to mesenchymal transition” concept, explained below [11,13].

### 2.1. Pancreatic Intraephitelial Neoplasia (PanIN)

PanINs are non-invasive microscopic epithelial neoplasms located in the smaller pancreatic ducts, characterized by architectural atypia [14]. PanINs are divided into three grades based on the degree of epithelial atypia, from only minimal atypia (PanIN-1) to marked atypia (PanIN-3) [15]. In addition, PanIN-1 lesions are further subdivided into flat (PanIN-1A) and papillary (PanIN-1B) types [16,17]. PanINs increase with age and are more common in the head than the tail of the pancreas [18]. PanINs are more common in pancreases with invasive carcinoma and in those with chronic pancreatitis [17]. A recent study suggests that morphologic changes in the pancreatic parenchyma adjacent to PanIN lesions may be detectable using currently available imaging technologies, such as endoscopic ultrasound: multifocal PanINs are frequently associated with a lobulocentric form of pancreatic parenchymal atrophy, which is detectable by ultrasounds [19].

As explained below, *KRAS* mutation is observed also in these pre-neoplastic lesions in codon 12 and exceptionally in codons 13 and 61 (75%–100%); the other *HRAS* and *NRAS* mutations were not reported in human PDAC [20]. HER-2/neu is not expressed in the epithelium lining of the normal pancreatic duct, but is highly expressed in PanIN (PanIN-1A: 82%, PanIN-1B: 86%, PanIN-2 and the higher grades: 92%) [21].

Abnormal loss of the *P16/CDKN2A* tumor suppressor gene is seen somewhat later than *KRAS* mutation and the frequencies are increased according to the progression of the grades of PanIN (PanIN-1A: 30%, PanIN-1B: 55%, PanIN-2 and the higher grades: 92%) [22].

In contrast, loss of P16 is less frequently observed in PanIN lesions arising in the background of chronic pancreatitis [23]. Loss of P16 function occurs via several different mechanisms, including homozygous deletion of *CDKN2A/INK4A*, intragenic mutation with loss of the second allele and epigenetic silencing by promoter methylation [24,25]. Alterations in TP53 and SMAD4/DPC4 tumor suppressors are absent in lower grade PanIN-1 or PanIN-2, but are observed in 12% and 30% of PanIN-3, respectively [26]. Immunohistochemical abnormal expression of TP53 and/or loss of immunostaining for SMAD4/DPC4 in PanIN may predict the progression of PanIN to PDAC, as these immunophenotypic changes correlate with the presence of alterations in the corresponding gene [26].

### 2.2. Intraductal Papillary Mucinous Neoplasms

The diagnosis and treatment of intraductal papillary mucinous neoplasms (IPMN) are of particular interest. In recent years it has become accepted that this combination of a diagnosable precursor of pancreatic cancers and its comparatively slow growth enable early diagnosis and curative surgical treatment [27]. The entity IPMN was included in the WHO classification system in 1996 [28]. Currently, IPMN account for 1%–3% of all exocrine pancreatic neoplasms and for 20%–50% of all cystic neoplasms of the pancreas [29]. The exact incidence of IPMN is not known however, because many of them are small and asymptomatic. There are no well-established etiological factors. In one series, most IPMN patients were cigarette smokers [30]. IPMN have been reported in patients with Peutz-Jeghers syndrome and in patients with familial adenomatous polyposis [31]. Some studies have suggested that IPMN may be particularly common among the neoplasms arising in patients with a history of familial pancreatic carcinoma (FPC) [32]. IPMN grow in the main duct or branch duct of the pancreas, produce mucin, and have differentiated papillary features. Since growths affecting the pancreatic main duct are associated with a higher malignant potential, IPMN are subcategorized clinically into main-duct (MD)-IPMN and branch-duct (BD)-IPMN [33].

The 2010 WHO classification subcategorizes IPMN according to their malignant transformation into IPMN, from low or intermediate to high-grade dysplasia and IPMN with invasive cancer. In addition to PanIN, IPMN are the most important precursor lesions of ductal pancreatic cancer. The non-invasive tumors have a much better prognosis than the invasive cancers, with a 5-year survival rate after resection of 90% compared with 60% [34].

Morphology and immunohistochemical staining with mucin antibodies enables differentiation between four types of tumor with different prognosis [28]: gastric, intestinal, pancreaticobiliary and oncocytic type. The intestinal type is most common: it usually occurs in the pancreatic head but may also involve the entire main duct, including the ampulla of Vater [35]; it shows a villous growth pattern similar to that of villous adenoma in the colon. It expresses Mucin-2 (MUC2), MUC5, and caudal type homeobox2 (CDX2), but not MUC1. For invasive intestinal IPMN, the invasive component corresponds to mucinous (colloid) carcinoma and is characterized by extensive stromal pools of extraluminal mucin, containing single cells or strands of neoplastic glandular epithelium or even a small component of signet ring cells [36,37]. The pancreatobiliary type also predominantly occurs in the main duct of the pancreas head, but it is much more rare than intestinal-type IPMN. It shows complex arborizing papillae and only expresses MUC1 and MUC5. When it becomes invasive, the invasive component usually corresponds to a conventional ductal (tubular) adenocarcinoma. The oncocytic type (also known as intraductal oncocytic papillary neoplasm) often forms large tissue nodules in the main pancreatic duct, with only little mucin production [38]. It shows the same complex papillae as the pancreatobiliary type, but the lining cells reveal strong eosinophilic cytoplasm and the presence of numerous goblet cells. The tumor cells focally and inconsistently express MUC1, MUC2, and MUC5AC. The gastric type mainly corresponds to branch duct (BD)-IPMN. It is probably the most frequent IPMN and is usually found in the periphery of the pancreatic parenchyma, most often in the uncinate process, where it presents as a multicystic lesion with cysts < 3 cm [39]. Histologically, it exhibits papillary projections lined by epithelial cells resembling gastric foveolar cells and shows pyloric gland-like structures at the bases of the papillae.

Activating *GNAS* mutations at codon 201 have recently been identified in IPMNs [40]. *GNAS* activating mutations were found in 64% of the IPMNs included in the study, and sub-analysis confirmed that demographic characteristics, tumor location, ductal system involvement, focality, size, grade of dysplasia, presence of an associated cancer, and overall survival were not correlated with *GNAS* mutational status. For these reasons, GNAS could become a promising target for early detection and therapy [41].

The reported frequency of *KRAS* mutations in IPMNs ranges from 38.5% to 100% [42]. There are two studies reporting that *P16/CDKN2A* inactivation increases along with the degree of dysplasia [43]. Aberrant hypermethylation has been shown in most IPMNs in at least one gene, and is associated with loss of gene expression such as *P16/CDKN2A* and *ppENK* [44]. Increase in the number of hypermethylated loci is related with increasing grade of dysplasia [45].

The identification of atypical cells by cytology in combination with a high CEA level in the cyst fluid was found to be more sensitive than the detection of malignant cells alone [46]. Monitoring as the approach for IPMN that does not require resection is contingent on the distinction between MD-IPMN and BD-IPMN. MD-PMN always constitutes an indication for surgery, whereas the treatment of BD-IPMN depends on clinical, morphological and imaging criteria. Asymptomatic BD-IPMN with a diameter <10 mm should be checked annually, and lesions of 10–20 mm every 6–12 months. In BD-IPMN >20 mm, the indication for surgery should be considered, depending on the clinical situation, and discussed individually with the patient. In the presence of symptoms, enlarged lymph nodes, a diameter >30 mm, or a dilated main duct, surgery is indicated. If no changes have occurred after two years’ monitoring, the interval may be extended. In case of indication for surgery, oncological pancreatectomy with lymphadenectomy should be performed. Tumor-free resection margins are required for all IPMN; if higher-grade dysplasia is found in the resection margin, then the resection should be continued until a negative margin has been achieved, even at the risk of total pancreatectomy [33]. There is no further evidence on adjuvant treatment for IPMN. Therefore, the role of adjuvant therapy in the management of IPMN remains unclear [47]. After resection of invasive IPMN, recurrence occurs in 40%–65% of patients, and lymph node involvement, vascular invasion, surgical margin involvement, and the presence of jaundice are adverse prognostic factors [27,48]. Several reports have demonstrated that, in terms of resectability, surgery is the only therapeutic option for recurrence, even for noninvasive IPMN [49].

### 2.3. Mucinous Cystic Neoplasms

Mucinous cystic neoplasms (MCNs) are defined as mucin-producing and septated cyst-forming epithelial neoplasia with a distinctive ovarian-type stroma without communication with the ductal system. Usually solitary, their size ranges between 5 and 35 cm with a thick fibrotic wall [50]. MCNs are rare, show a female to male ratio of 20:1 and a mean age at diagnosis of between 40 and 50 years (range 14–95 years). The site of the neoplasm is in the body and tail of the pancreas in 98% of cases [29].

Macroscopically, MCNs usually appear as solitary, multilocular or unilocular lesions with a mean size of 7–8 cm (range 0.5–35 cm). They have a thick fibrotic wall and contain mucin, even when hemorrhagic watery or necrotic content is observed [51]. Light microscopy reveals that the cysts are lined by a columnar mucin-producing epithelium with different grades of dysplasia: mild (MCN adenoma), moderate (MCN borderline) and severe (MCN carcinoma *in situ*) [52]. The epithelial lining is positive for CKs (CK7, CK8, CK18, CK19), EMA and, less frequently, CK20, CEA, DUPAN-2 and CA 19-9 [53].

*KRAS* mutation at codon 12 is observed as the early event and the frequency increases according to the degree of dysplasia. On the other hand, *TP53* mutation is a relative late event in *in situ* or invasive mucinous cystic-adenoma-carcinomas [54,55]. Inactivation of the *SMAD4/DPC4* gene is also a late event, and about half of invasive mucinous cystadenomacarcinomas show loss of Dpc4 expression. Benign MCNs, however, show no loss of expression [15]. It is very interesting that ovarian-type stromal cells show no loss of Dpc4, suggesting non-neoplastic characteristics of the stromal cells. The immunophenotype of ovarian-type stroma is similar to the normal ovarian one with positivity for vimentin, calretinin, tyrosine hydroxylase, SMA, α-inhibin, Melan-A, CD99 and Bcl-2 and frequently for progesterone and estrogen receptors. The origin of ovarian stroma of the pancreas is still being debated [56]. A stimulation of endodermal immature stroma by female hormones or primary yolk cell implantation in the pancreas has been suggested, because buds of the genital tract and dorsal pancreas are adjacent to each other during embryogenesis. Moreover, dorsal pancreatic enlargement mainly gives rise to the pancreatic body and tail, and this could explain the predilection of MCNs for the distal pancreas [57].

The majority of MCNs are slow growing and asymptomatic and are occasionally discovered in patients scanned for other indications, such as epigastric heaviness and fullness (60%–90%) or an abdominal mass (30%–60%) [58]. Nausea, vomiting (20%–30%) and back pain (7%–40%) can also be present. Increasing anorexia and weight loss (10%–40%) may be associated with malignant changes [59]. Patients with invasive mucinous cystadenocarcinoma show a 5-year survival rate of 20%–60%, which is much better than that for non-MCN-associated ductal adenocarcinoma. When an anaplastic carcinoma of the pancreas associated with MCN is reported, the prognosis is extremely poor, with a 3-year survival rate lower than 3% [60].

## 3. Activated Pathways and Altered Processes in Pancreatic Ductal Adenocarcinoma

Oncogenes are genes that contribute to oncogenesis when mutationally activated. In this mutation setting, only one copy of the gene suffices for activation. Oncogenes can be activated through a variety of mechanisms (point mutations, amplification). A growing number of oncogenes have been identified during recent years that are targeted in pancreatic cancer.

The most common activating point mutation involves the *KRAS* oncogene. This mutation (chromosome 12) is present in over 90% of PDAC [61,62], and involves the highest fraction of KRAS alteration found in any human tumor type. The most frequent mutation sites involve codon 12. Oncogenic *KRAS* mutations are observed in early pancreatic lesions as previously described [63]. The central role of KRAS in the activation of many cellular activities explains how much the constitutive activated state of the protein determines a great gain of function, which leads to proliferation, suppressed apoptosis and cell survival. The constitutively active *RAS* confers uncontrolled stimulatory signals to downstream cascades including Ras effectors, notably the *RAF*-mitogen-activated protein kinase, phosphoinositide-3-kinase (PI3K) and RalGDS pathways [64]. Mutant KRAS has been extensively investigated as a marker of pancreatic cancer because mutations are basically entirely limited to one codon, and can be readily detected using molecular assays. Unfortunately, *KRAS* mutations are not specific to invasive pancreatic cancer and also occur in patients with chronic pancreatitis or *in situ* neoplasias, or in smokers. [65,66]. One potential target studied for therapy is inhibition of farnesyltransferase, which adds a hydrophobic 15-carbon isoprenoid chain to the cysteine residue of *KRAS*, allowing it to anchor to the cell membrane. Preclinical studies showed that farnesyltransferase inhibitors (*FTI*) affect cell growth and cell cycle regulation involving the post-translational processing of Harvey rat sarcoma (*HRAS*) and neuroblastoma rat sarcoma (*NRAS*) [67], and currently preliminary clinical data are available about target drugs used in PDAC patients, such as tipifarnib [68] and salirasib [69].

Another member of the *RAS* cascade is the *BRAF* gene, signed on chromosome 7q, a serine/threonine kinase involved in the *RAS-RAF-MEK-ERK-MAP* kinase pathway. Its mutation frequency in PDAC with wildtype KRAS is 30% [54]. Interestingly, all studies in recent years have noted that *KRAS* and *BRAF* mutations are mutually exclusive and tumors with mutant forms of one of these 2 genes invariably retain wild-type copies of the other. The requirement of the oncogenic KRAS or BRAF pathway appears to be critically important for most instances of pancreatic ductal carcinogenesis, and for this reason studies evaluating these oncogenes as potential target therapy are warranted.

The *PI3K*-kinase-*AKT* pathway is a key effector of *RAS*-dependent transformation of many cell types and also plays a role in cell survival and other growth-related processes [70]. Activated *PI3K* results in phosphorylated phosphatidylinositides (*PIP3*), a step inhibited by product of the tumor suppressor gene, *PTEN*. Activating mutations of *PIK3CA*, the gene encoding *PI3K*, have been reported in a subset of pancreatic cancer precursors, specifically in IPMNs [71]. The *PI3K/AKT* pathway is constitutively active in the majority of pancreatic cancers [72]. Another downstream pathway activated through *RAS* is the *RalGDS* pathway, one of several known Ras-regulated guanine-nucleotide exchange factors, or *GEFs*, that function by activating *Ral A* and *Ral B* GTPases [73]. *RAL A* is activated in a variety of pancreatic cancers, and knockdown of *RAL A* suppressed tumorigenicity and metastases of RAS-transformed human cells [74].

Previous mouse studies showed that pancreatic deletion of PTEN or expression of constitutively active AKT leads to expansion of central acinar cells, putative pancreatic progenitors, and formation of PDAC in a small percentage of mice, supporting the role of PTEN in PDAC development [75,76]. The group of Bardeesy documented in their study strong cooperative interactions of KrasG12D and PTEN loss in promoting metastatic PDAC. Mouse PDAC driven by oncogenic Kras mutation and PTEN deficiency also sustain spontaneous extinction of Ink4a expression and show prometastatic capacity. Thus, the PTEN/PI3K pathway alteration is recognized as a common event in PDAC development and functions in part to strongly activate the NF-κB network, which may serve to shape the PDAC tumor microenvironment [77].

The hedgehog signaling pathway is another crucial system involved in the early invasion and metastatic spread of pancreatic cancer cells. It is activated by two transmembrane proteins, the patched (*PTCI*), a tumor suppressor, and smoothened (*SMO*), an oncogenic protein [78]. Activation of the hedgehog pathway has been implicated in both the initiation and the maintenance of advanced cancers. Several preclinical studies showed that hedgehog ligands are overexpressed in PDAC (above 70% of all cases) [79–83]. The former may promote the formation of desmoplastic stroma, an important component of the tumor microenvironment, and hinder effective drug delivery [83]. By targeting the tumor microenvironment and cancer stem cells, hedgehog inhibitors could potentially not only improve drug delivery to malignant cells but also diminish further systemic metastasis. The Sonic Hedgehog ligand (Shh), absent in normal pancreas, is highly expressed in pancreatic tumors and is sufficient to induce neoplastic precursor lesions in mouse models. Hebrok’s group investigated the mechanism of Shh signaling in PDAC carcinogenesis by genetically ablating the canonical bottleneck of hedgehog signaling, the transmembrane protein *SMO*, in the pancreatic epithelium of PDAC-susceptible mice. They observed that autocrine Shh-Ptch-Smo signaling is not required in pancreatic ductal cells for PDAC progression, highlighting an independent mechanism of progression, necessary for further analysis [84].

Preclinical trials explored *SMO* hedgehog inhibitors, such as cyclopamine, which were able to limit pancreatic cancer metastasis in a spontaneously metastasizing xenograft model and to influence chemoresistance to gemcitabine in pancreatic cancer cells [85]. Recently, another *SMO* inhibitor, saridegib (IPI-926), more potent than cyclopamine, yielded a wide depletion of desmoplastic stroma and a decrease in collagen I levels with respect to the gemcitabine control in a preclinical trial [86]. Interestingly, saridegib did not affect cellular proliferation of the pancreatic cancer cells, confirming a predominant effect on peritumoral stroma [86]. These data provide the rationale to combine this hedgehog inhibitor with cytostatic drugs. Further investigation of this class agent is recommended.

The Notch signaling pathway is another pathway that is important in directing cell fate and cell proliferation during embryonic development. It plays a critical role in maintaining the balance among cell proliferation, differentiation, and apoptosis [87]. The function of Notch signaling in tumorigenesis can be either oncogenic or antiproliferative, and the function is context dependent. In a limited number of tumor types, including human hepatocellular carcinoma and small cell lung cancer, Notch signaling is antiproliferative rather than oncogenic. However, most of the studies show an opposite effect of Notch in many human cancers including PDAC [88]. In the normal adult pancreas, Notch and its ligands are expressed at low levels. Interestingly, aberrant expression of its ligands together with expression of the mutant Notch1 oncoprotein can be observed in early stages of pancreatic tumorigenesis and are more represented in invasive pancreatic cancer [89].

**Figure 2:**
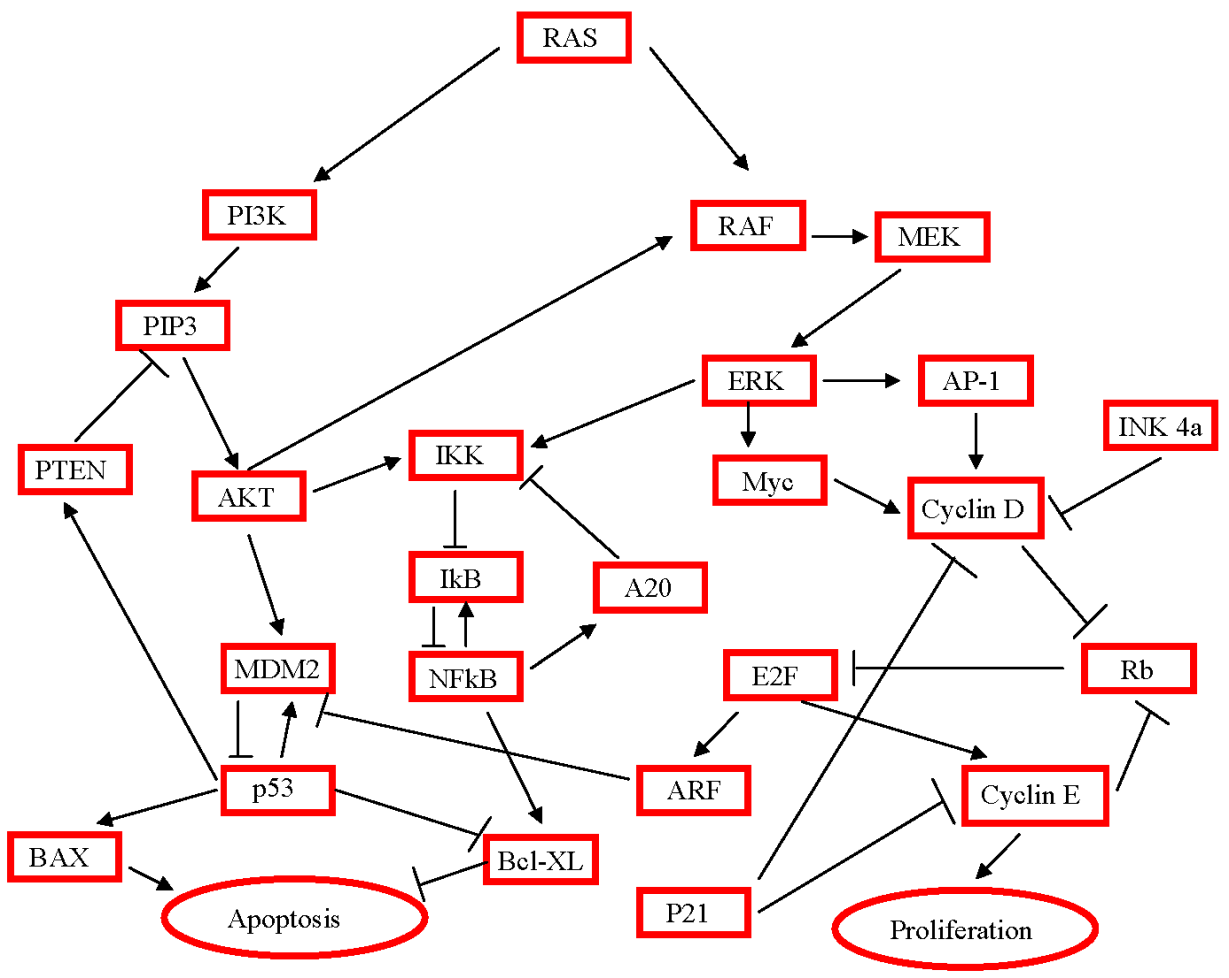
Interaction between pathways to regulate pancreatic carcinogenesis.

Several other oncogenes that are targeted in pancreatic cancer by amplifications deserve mentioning. The *AKT2* gene on chromosome 19q is a downstream effector of the *PI3K/AKT* pathway, and is amplified in 10%–15% of pancreatic cancers [90]. *AKT2* can be activated by stimuli such as platelet-derived growth factor, basic fibroblast growth factor, and insulin through the *PI3K/AKT* pathway, suggesting this pathway’s importance in this tumor type [91]. The *MYB* gene on chromosome 6q is amplified in 10% of pancreatic carcinomas [92]. Abnormalities in the locus of the human *MYB* gene have been observed in several human cancers. In a majority of these tumors, these abnormalities seem to be accompanied by an amplification of the *MYB* gene followed by enhanced transcription [92]. Figure 2 shows all pathways and their interactions involved in PDAC carcinogenesis.

As regards oncogene mutations, besides genetic alterations, epigenetic mechanisms for gene inactivation such as transcriptional silencing by promoter methylation seem to be equally important in the pathogenesis of PDAC [93]. The activity of these factors determines the silencing of tumor-suppressor and cancer-related genes in pancreatic cancers, among them BRCA1, APC, and p16INK4a [94].

These biological changes used for the detection of PDAC should ideally be present early on in pancreatic carcinogenesis and precancerous lesions. Due to the disease’s rapid progression and early metastasis formation, these suppressor genes may be deregulated early during pancreatic carcinogenesis and acquire the concept of metastases suppressor genes (*MSGs*). Tumor suppressor genes are genes that promote tumor growth when inactivated.

The *P16INK4A/CDKN2A* gene, located on the short arm of chromosome 9 (9p), is one of the most frequently inactivated tumor suppressor genes in pancreatic cancer [24]. Virtually all pancreatic carcinomas have loss of P16INK4A/CDKN2A function; in 40% of pancreatic cancer cases there is a homozygous deletion. In the same percentage there is an intragenic mutation coupled with loss of the second allele, and in 15% of cases cancer occurs after a hypermethylation of the *P16INK4A/CDKN2A* gene promoter [24]. The protein p16 belongs to the cyclin-dependent kinase (*CDK*) inhibitor family and functions to prevent the phosphorylation of several cyclins, and cell-cycle regulators [95]. Loss of P16INK4A/CDKN2A results in inappropriate phosphorylation of *Rb-1*, thereby facilitating progression of the cell cycle through the G1/S transition [95]. In a small group of patients, inherited mutations of the *P16INK4A/CDKN2A* gene cause familial atypical multiple mole melanoma (FAMM) syndrome, which is associated with an increased risk of developing melanoma and PDAC [96].

Mutation of the *p53* gene on chromosome 17p is the most common somatic alteration in human cancer. The *p53* protein plays a central role in modulating cellular responses to cytotoxic stress by contributing to both cell cycle arrest and programmed cell death. Loss of *p53* function during carcinogenesis can lead to inappropriate cell growth, increased cell survival, and genetic instability [97]. In pancreatic cancer, the p53 tumor suppressor gene is inactivated in 50%–75% of cases and occurs predominantly through single allelic loss coupled with an intragenic mutation of the second allele [98]. The loss of p53 means that two critical controls of cell number (cell division and cell death) are deregulated in the majority of PDAC. In addition, p53-induced growth arrest is also achieved by transactivation of p21. p53 binding to DNA stimulates production of the protein p21, which negatively regulates the complex consisting of cyclin D and the cell division-stimulating protein cyclin-dependent kinase-2 [99], allowing time for repair to damaged DNA. If p53 mutates, it is not able to bind DNA, so p21 is not made available and abnormal growth can occur. Cell lines that lack wild-type p53 show a reduced or complete absence of p21 [99]. Loss of p21 activity has been observed in approximately 30%–60% of pancreatic tumor specimens [100].

*DPC4* (*Smad4*) is a tumor suppressor gene on chromosome 18q and is one of the most commonly inactivated genes in PDAC, detected in approximately 55% of cases. Inactivation occurs either through homozygous deletion, in approximately 30%, or loss of one allele coupled with an intragenic mutation in the second allele in approximately 25% [26,101]. The transcription factor SMAD4 is an important regulator of the transforming growth factor-β (*TGF-*β) signaling pathway. Upon receptor activation, SMAD proteins become phosphorylated and heterodimerize with Smad4 to transmit upstream signals to the nucleus and transactivate transcription of specific target genes [102]. Loss of SMAD4/DPC4 interferes with the intracellular signaling cascades downstream from *TGF-*β and activin, resulting in decreased growth inhibition via loss of proapoptotic signaling or inappropriate G1/S transition [102]. The *SMAD4* gene is notable primarly for the reason that immunohistochemical labeling for SMAD4 protein expression mirrors *DPC4/SMAD4* gene status in pancreatic cancers with rare exceptions [26]. Therefore, immunolabeling for loss of Smad4 can be a convenient ancillary diagnostic marker in clinical specimens, including suspected metastases from an occult pancreatic primary.

Furthermore, *TGF-*β is a pleiotropic factor that regulates cell proliferation, angiogenesis, metastasis, and immune suppression. The involvement of the *TGF-*β pathway has been established in cancers of many organs including the breast, lung, colon and pancreas. *TGF-*β signaling is frequently attenuated in PDAC because of alterations in the components of the pathway [103]. Further data are mandatory in this setting, especially in other tumor suppressor genes that in recent years have been evaluated in several cancer cells and may contribute to adding other pieces to the complex mosaic of factors responsible for tumor initiation and development.

Genome maintenance genes are those that function to identify and repair damage to DNA. When this regulatory system is inactivated, DNA damage is not repaired efficiently and DNA mutations accumulate. These mutations in cancer-associated genes contribute to tumorigenesis [104]. Genetic instability also occurs through DNA mismatch repair defects in PDAC [105]. The DNA mismatch repair system consists of at least six genes: *hMLH1*, *hMSH2*, *hMSH3*, *hMSH6*, *hPMS1*, and *hPMS2*. Of these, *hMLH1* and *hMSH2* are examples of genome maintenance genes targeted in pancreatic cancer [106]. When one of these genes is inactivated, DNA changes occur leading to the phenomenon named “microsatellite instability” (MSI). MSI is associated with aggressive disease (poor differentiation) and other genetic changes, such as lack of *KRAS2* and *p53* mutations. Approximately 4% of pancreatic cancers have MSI and these cancers have a typical microscopic appearance called “medullary type”, with a syncytial growth pattern, pushing borders and lymphocytic infiltrate [106]. Marcus *et al*. used immunohistochemistry to identify MSI from the expression of *hMLH1* and *hMSH2* genes. The sensitivity and specificity of the test was 97% and 100%, respectively [107]. The use of immunohistochemistry offers a relatively rapid method for prescreening tumours for defects in the expression of *MMR* genes. Tomaszewska *et al.* showed that the presence of endocrine cells in PDAC is a frequent phenomenon, and it was significantly associated with expression of the *hMSH2* gene [108].

Other maintenance genes playing a role in pancreatic tumorigenesis are genes of Fanconi anemia, FANCC and FANCG [109]. This disease is a hereditary cancer susceptibility disorder, with hematologic abnormalities at an early stage, usually leading to death before the age of 20. Patients who survive into adulthood often develop solid tumors [110]. Both mutations (*FANCC* and *FANCG*) were associated with loss of heterozygosity of the wild-type allele in corresponding pancreatic tumors. The previously described *BRCA2* gene represents Fanconi complementation group D1 and is thought to aid DNA strand and interstrand crosslinking repair. For this reason BRCA2 can be categorized as a genome maintenance gene. In PDAC 7%–10% harbor an inactivating intragenic inherited mutation of one copy of the *BRCA2* gene, accompanied by loss of heterozygosity [111]. Gallmeier *et al.* experimented using endogenous disruption of FANCC and FANCG in a human PDAC cell line and determined the impact of these genes on drug sensitivity, irradiation sensitivity, and genome maintenance. On treatment with DNA interstrand-cross-linking agents, FANCC and FANCG disruption caused increased clastogenic damage, G2/M arrest, and decreased proliferation. Also a major chemosensivity to melphalan and oxaliplatin was highlighted, while no increased response was observed when authors tested gemcitabine or etoposide [112]. FANCC and FANCG disruption also resulted in increased clastogenic damage on irradiation and increased spontaneous chromosomal breakage, supporting the role of these genes in genome maintenance and likely explaining why they are mutated in sporadic cancer. The lack of response to gemcitabine treatment has been the limiting factor that has held back subsequent studies in this setting. However, irradiation sensivity still remains the way forward for the future. Tables 1 and 2 summarise the oncogenes and tumor suppressor genes in PDAC with genetic aberration percentage and types.

## 4. Epithelial to Mesenchymal Transition in Invasion and Metastasis

Epithelial-to-mesenchymal transition (EMT) is a collection of events that allows the conversion of adherent epithelial cells, tightly bound to each other within an organized tissue, into independent fibroblastic cells possessing migratory properties and the ability to invade the extracellular matrix [113]. Physiologically, EMT contributes to the complex architecture of the embryo by permitting the progression of embryogenesis from a simple single-cell layer epithelium to a complex three-dimensional organism composed of both epithelial and mesenchymal cells. Normally, in most tissues EMT is a developmentally restricted process. Over the last few years, elements of EMT, especially the loss of epithelial markers and the gain of mesenchymal markers, have been observed in pathological states, including epithelial cancers [114].

Increasing evidence has confirmed its presence in carcinogenesis and chronic inflammation processes. The cells also develop a mesenchymal phenotype, taking on a spindle-like, fusiform morphology, become motile, and start expressing mesenchymal markers, e.g., *N*-cadherin, fibronectin, and vimentin [115]. A large body of evidence supports roles for several signaling pathways, such as macrophage migration inhibitory factor (*MIF*), *SMAD/STAT3*, the *NF-*κ*B* pathway, *Ras*-mitogen-activated protein kinase/*Snail/Slug* and microRNAs [116]. Thus, EMT appears to be closely involved in the pathogenesis of PDAC, and analysis referred to it can yield novel targets for therapy. There is increasing evidence of the contribution of EMT to pancreatic cancer metastasis and to treatment resistance.

The key regulators of EMT include *Snail*, *Slug*, *Zeb1*, and *Twist*, which are zinc finger transcription factors that repress genes responsible for the epithelial phenotype [116]. These factors are associated with decreased *E*-cadherin expression (epithelial factor), increased migration and invasion, higher tumor grade and worse outcomes. Transforming growth factor-β (*TGF-*β), one of the primary drivers of EMT, can increase expression of *Snail*, *Slug* and *Zeb1* in PDAC [115]. EMT is also associated with cancers becoming oncogene independent. This process can bypass oncogene activation via *K-Ras* [117]. Inflammation factors also play a significant role in PDAC through *NF-*κ*B*, increasing both EMT and cancer cell invasion [118]. *Snail* activity is increased via stabilization at the protein level in response to *TNF-*α driven *NF-*κ*B* signaling. Interestingly, *TGF-*β-induced EMT is also dependent on *NF-*κ*B* signaling [119]. So NF-κB, as a master regulator of innate immunity and inflammation, represents a molecular bridge between chronic inflammation and cancer development. Also the functions mediated by NF-κB are at least partially carried out in cooperation with other factors such as the signal transducer and activator of transcription 3 (STAT3), that represents a critical component of pancreatitis-accelerated PanIN formation and supports cell growth and metaplasia-associated inflammation. So NF-κB and STAT3 in PDAC cells cooperate in a signalling dependent manner promoting cellular functions associated with pancreatic cancer development and progression [120].

MIF, a lymphokine involved in cell-mediated immunity and inflammation, is implicated in cancer [121]. Gene expression profiling of PDAC revealed an overexpression of MIF, as well as regulation of cellular signal transduction [122]. Higher MIF levels are found in many human cancers and inflammatory diseases, including chronic pancreatitis and PDAC. MIF expression was also found to relate to the degree of cell differentiation of PDAC [123]. Consistent data have shown that elevated MIF mRNA expression in tumors was significantly associated with poor outcome in resected cases, with an independent association with patient survival (HR = 2.26, *p* = 0.015) [124]. Mechanistic analyses revealed that MIF overexpression increased protein levels in pancreatic cancer cell lines, consistent with the features of EMT. These results support a role of MIF in disease aggressiveness, indicating its potential usefulness as a candidate target for designing improved treatment in pancreatic cancer [124].

EMT has also been shown to be a significant contributor to chemo-resistance in several cancers, including PDAC [125,126], as evidenced in gene expression profiling. Specifically, the EMT transcription factor *Zeb1* is upregulated in resistant cell lines and correlates with decreased expression of E-cadherin. Significantly, maintenance of chemoresistance in cell lines that have undergone EMT is dependent on *Notch* and *NF-*κ*B* signaling [127]. EMT plays a role in modulating resistance not only to traditional chemotherapies, but to targeted biologic therapies as well. Cells that express either mutated E-cadherin, or have high levels of *Snail*, *Zeb1*, and vimentin, and thus a mesenchymal phenotype, show significantly decreased growth inhibition in response to treatment with the EGFR inhibitor erlotinib than cells with an epithelial phenotype [128]. All this evidence supports the concept that pronounced fibrotic reaction, primarily generated by mesenchymal elements, such as myofibroblast-like stellate cells, can limit the delivery of current chemotherapeutic agents to the cancer cells [129]. For this reason, the chemoresistance of pancreatic cancer can be a dynamic process acquired during evolution and therapy response. Given the role of EMT in chemo-resistance and tumor progression, specifically targeting EMT could improve the survival rates of pancreatic cancer patients. Currently clinical trials targeting *Zeb1* and *Slug* expression (the naturally occurring flavanoid Silibinin) [130], and Hedgehog, *Wnt* and *Notch* signaling, known EMT pathways that have been implicated in cancer stem cells and chemoresistance [126], are also underway.

## 5. Epigenetic

### 5.1. Chromatin-Based Epigenetics

It is recognized that DNA hypermethylation at gene promoter CpG islands contributes to tight transcriptional repression of many genes in human cancer; the frequency of the CpG dinucleotide in the human genome is lower than expected for the spontaneous deamination in the germline during evolution [131]. However, approximately half of the human gene-promoter regions contain CpG-rich regions with lengths of 0.5 to several Kb. It should also be noted that although the most significant proportion of CpG islands is located in the 5′-untranslated region and the first exon of the genes, certain CpG islands are occasionally found within the body of the gene, or even in the 3′-region. CpG islands in these atypical locations are more prone to methylation [131], and the RNA transcript can cross over them without any evident impediment [132]. Exceptionally, certain small genes can be considered in their totality as a whole CpG island. Typical CpG islands are entirely unmethylated at all stages of development and allow the expression of a particular gene if the appropriate transcription factors are present and the chromatin structure is accessible to them. In the transformed cell, certain CpG islands of tumor-suppressor genes will become hypermethylated. Although the majority of these are associated with “house-keeping” genes, half of the “tissue-specific” genes also contain a promoter CpG island [133]. The questions of which and how DNA methylation changes in tissue-specific genes occur in cancer remain largely unanswered. This epigenetic silencing constitutes an alternative to genetic mechanisms that mediate loss of function for many tumor-suppressor genes [134].

Schlesinger *et al.* showed that, in colon cancer, DNA hypermethylation is mediated by the presence of H3K27Me3; however, Ohm *et al.* and Widschwendter *et al.* both demonstrate the strong association between genes with *H3K27Me3* and DNA hypermethylation: it was found that many genes with *de novo* promoter hypermethylation in colon cancer were among the subset of genes marked in embryonic cells by repressive Polycomb group proteins (PcG), in the context of “bivalent” chromatin that, in the embryonic system, occurs in non-DNA-methylated promoter CpG islands and consists of the simultaneous presence of the repressive PcG mark (H3K27Me3) and the active transcription marks H3K4Me2/Me39 [135]. Such chromatin is thought to maintain low, but poised, transcription of genes that otherwise upon active transcription would cause lineage commitment and disruption of stemness and the self-renewal status of embryonic stem cells [136,137].

Cancer cells possess hallmarks of embryonic stem cells: the capacity for self-renewal and an undifferentiated cell state [138,139], which are a fundamental property of most tumorigenic, and often therapy-resistant, subpopulations of cells in human cancers [140,141]. However, most human cancers are not derived from embryonic cells, and the relationship between cancer and adult cell renewal systems has been less clearly described. Easwaran *et al.* show that the methylation status of most genes can cluster important subtypes of colon and breast cancers and, by evaluating the subsets of genes that are hypermethylated in different cancers, they provide evidence that DNA hypermethylation preferentially targets the subset of *PcG* genes that are developmental regulators, and this may contribute to the stem-like state of cancer [142]. More studies showed that age-related methylation of specific CpG islands, which also get methylated in cancers, targets genes marked by PcG in stem cells [143]. Thus, the mechanisms underlying the selective targeting of a subset of PcG-marked genes seem to operate during normal physiology and disease; however, more studies are needed. The selective advantage to tumors may arise from cumulative silencing of a group of developmental regulators rather than individual genes. In particular, in pancreatic cancer, Neuronal pentraxin II (NPTX2) has been observed to be hypermethylated; thus, methylation of NPTX2 might provide a novel diagnostic marker for pancreatic cancers. In the study of Zhang *et al.*, NPTX2 expression was detected by RT-PCR and the methylation status was assessed by methylation-specific polymerase chain reaction. The pancreatic cancer cell lines were treated with DNA methyltransferase inhibitors or histone deacetylase inhibitors. Analysis revealed that the promoter region of the *NPTX2* gene was largely unmethylated in normal pancreatic tissues, while NPTX2 was frequently hypermethylated in pancreatic cancer cells and in primary pancreatic carcinomas. Quantitative RT-PCR revealed that the mean mRNA expression level of NPTX2 in the pancreatic cancer tissues was significantly lower than that in the paired adjacent normal tissues (0.96 ± 0.91 *vs*. 2.78 ± 1.42, *p* < 0.001). This study provides the first evidence that the down-regulation of NPTX2 tightly correlates with its promoter hypermethylation [144].

A study by Aghdassi *et al.* investigated how E-cadherin expression in human pancreatic adenocarcinoma and pancreatic cancer cell lines is regulated (loss of the cell adhesion molecule E-cadherin is frequent during epithelial-mesenchymal transition and can be caused by genetic or epigenetic modifications). In 25 human pancreatic cancer resection specimens, the coding region of the *E-cadherin* gene (*CDH1*) was sequenced for somatic mutations. The role of specific histone deacetylase inhibitors (HDACi) on pancreatic tumour cell migration and proliferation was studied *in vitro*. Expression of ZEB1, a transcription factor known to recruit HDACs, was seen in E-cadherin-deficient cell lines; moreover, knockdown of ZEB1 prevented HDAC from binding to the CDH1 promoter, resulting in histone acetylation and expression of E-cadherin. HDACi treatment attenuated tumour cell migration and proliferation. Recruitment of HDACs to the CDH1 promoter is regulated by the transcription factor ZEB1, and inhibition of HDACs may be a promising antitumour therapy for pancreatic cancer [145]. The study of Cai *et al.* assessed the status of methylation in the CpG island of the tumor necrosis factor receptor superfamily member 10c (TNFRSF10C) with combined bisulfite restriction analysis (COBRA), and evaluated its role in the progression of pancreatic cancer. Changes in methylation and TNFRSF10C expression in pancreatic cancer cell lines before and after treatment with 5-aza-2′-deoxycytidine (5-aza-dC) and/or trichostatin A (TSA) were assessed. After treatment with 5-aza-dC and/or TSA, apoptosis was induced in pancreatic cancer cells to different degrees, and the levels of TNFRSF10C transcriptional expression in the pancreatic cancer cell lines increased markedly after 5-aza-dC treatment [146].

### 5.2. MicroRNA

MicroRNAs (miRNAs), which were discovered in *Caenorhabditis elegans* in 1993, have revealed new mechanisms for the regulation of gene expression and have provided new directions for cancer research. MiRNAs belong to a family of highly conserved, noncoding, 17–25 nucleotide3-long RNA products that regulate gene expression at the post-transcriptional level [147]. They are negative regulators of gene expression that functioned primarily through imperfect base pair interactions with sequences within the 3′ untranslated region of protein-coding miRNAs; their biosynthesis is a multi-step process, involving nuclear and cytoplasmic components. A number of approaches have been described to quantify miRNAs levels [148,149]: these approaches revealed distinct cell- and tissue-specific miRNA expression in pancreatic cancer specimens as compared with other normal cells and tissues. An early reported application of real-time PCR profiled more than 200 miRNAs precursors in specimens of human PDAC, paired benign tissue, and normal pancreas. One hundred miRNAs precursors were aberrantly expressed in pancreatic cancer or desmoplasia (miR-155, miR-21, miR-221, miR-222, miR-376a,s and miR-301). Mature miRNAs showed that three of the top differentially expressed miRNAs (miR-221, miR-376a, and miR-301) were localized to tumor cells and not to stroma, normal acini, or ducts [150].

The mechanism of action of a specific miRNA usually involves nucleotide complementary nucleotide pairing to the 3′UTR of its specific target miRNA, where it primarily functions as a negative regulator by repressing target miRNA translation [150]. Genes targeted by miRNAs are highly enriched and play a crucial role in regulating apoptosis, proliferation, migration, and invasion of PDAC cells. These functions of miRNAs in pancreatic cancer can affect the prognosis of patients.

Roldo *et al*. showed that a common pattern of miRNA expression distinguishes any tumor type from a normal pancreas. For example, miR-204 is primarily expressed in insulinomas and correlates with immunohistochemical detection of insulin, and the overexpression of miR-21 is strongly associated with both a high Ki67 proliferation index and the presence of liver metastasis [151]. Bloomston *et al*. demonstrated that up-regulation of miR-155, miR-181, miR-21, miR-196a and miR-221 and down-regulation of miR-148 and miR-375 differentiated pancreatic cancer from normal pancreas and pancreatitis tissue samples. Moreover, miR-196a expression was up-regulated and its levels inversely correlated with survival in PDAC patients [152,153]. Others miRNAs most frequently reported in the literature with aberrant expression in PDAC were mir-15b, miR-146a, miR-200 and miR-221/222 [154]. Habbe *et al*. reported that miR-155 and miR-21 are also overexpressed significantly in tissue from IPMNs [155]. MiR-34a is a significant component of the TP53 transcriptional network and during DNA damage, and is commonly deleted in human cancers such as PDAC. MiR-96 is considered a potential tumor suppressor (directly targets and down-regulates the *KRAS* oncogene): in PDAC, it is significantly down-regulated when compared with normal pancreatic tissues; in human clinical specimens, an inverse correlation was observed between miR-96 and KRAS expression (miR-96 may have potential therapeutic use in KRAS-driven pancreatic cancer) [156].

MiRNAs appear also to be involved at several points along the tumor’s pathway to acquisition of migratory and invasive properties. The already mentioned miR-21 targets phosphatase and tensin homologue 2 (PTEN), programmed cell death 4 (PDCD4), trophomyosin 1 (TPM1), and tissue inhibitor of metalloproteinases 3 (TIMP3), leading to inhibition of apoptosis and consequent increased tumorigenicity [157,158]. Re-expression of miR-146a inhibited the invasive capacity of PDAC cells with concomitant down-regulation of EGFR and the NF-κB regulatory kinase interleukin 1 receptor-associated kinase 1(IRAK-1) [159]. In another study, manipulation of miR-31 expression led to reduced cell migration and invasion in pancreatic cancer [160]. A variety of miRNAs have been shown to induce changes in the chemosensitivity or radiosensitivity of pancreatic cancer cells. The cells that overexpress miR-21 precursor show increased chemoresistance to gemcitabine compared with control cells [161].

In other studies, transfection of the synthetic miRNA (Gli-1-miRNA-3548) and its corresponding duplex (Duplex-3548) significantly inhibits Gli-1, leading to the inhibition of proliferation, delayed cell division, and activation of late apoptosis in MIA-PaCa-2 cancer cells [162]; miR-96 directly targets the *KRAS* oncogene, and ectopic expression of miR-96 can reduce pancreatic cell proliferation, migration, and invasion, indicating its potential therapeutic role in pancreatic cancer. These miRNAs with oncogenic or tumor suppressor functions (let-7, miR-21, miR-27a, miR-31, miR-200, and miR-221) could be used as novel therapeutic agents for pancreatic cancer [163,164]. Antisense to miR-21 and miR-221 sensitized the effects of gemcitabine, and the antisense-gemcitabine combinations were synergistic at the high fractions affected [165]. Iwagami *et al.* demonstrated that miR-320c induce resistance to gemcitabine in gemcitabine-resistant clones of MiaPaCa2 (MiaPaCa2-RGs); further experiments showed that miR-320c-related resistance to gemcitabine was mediated through SMARCC1, a core subunit of the switch/sucrose nonfermentable (SWI/SNF) chromatin remodeling complex. In addition, clinical examination revealed that only SMARCC1-positive patients benefited from gemcitabine therapy with regard to survival after recurrence (*p* = 0.0463), suggesting that miR-320c/SMARCC1 could be suitable for prediction of the clinical response and potential therapeutic target in pancreatic cancer patients on gemcitabine-based therapy [166]. In the study of Mace *et al.*, MiR-21 levels increased in all cell lines grown in hypoxic conditions versus normoxia, whereas miRNA targeting HIF-1α reduced miR-21 expression. Hypoxic conditions resulted in direct binding of HIF-1α to the predicted binding site in miR-21. Transfection with a constitutively stable HIF-1α expression plasmid in normoxia resulted in upregulated miR-21, similar to that seen in hypoxia. Cells transfected with antisense constructs targeting miR-21 had reduced proliferation and increased apoptosis in normoxia, whereas miR-21 overexpression abrogated hypoxia-associated reductions in proliferation. MiR-21 is induced by hypoxia in pancreatic cancer cells via HIF-1α upregulation. MiR-21 overexpression allows cells to avoid apoptosis in a hypoxic microenvironment; inhibition of miR-21 expression may increase cellular susceptibility to hypoxia in pancreatic cancer [167].

Let-7 expression was repressed in patients with PDAC who were not eligible for surgery: restoring let-7 levels in cancer-derived cell lines strongly inhibits cell proliferation, Kras expression, and mitogen-activated protein kinase activation, but fails to impede tumor growth progression after intratumoral gene transfer or after implantation of Capan-1 cells stably overexpressing let-7 miRNA [168].

## 6. Tumor Microenvironment: Role in Carcinogenesis and Therapeutic Potential

Most conventional and targeted therapies fail to provide substantial response rates in pancreatic cancer. The challenges faced by oncologists in the treatment of pancreatic cancer may in part be explained by the diverse influences exerted by the tumor microenvironment (TME). The molecular mechanisms of the microenvironment-tumor cell cross-talk are challenging due to the heterogeneous nature of the PDA stroma compared to other neoplasms [169]. Feig *et al.* in their review assessed that PDAC is one of the most stroma-rich cancers and comprises several cellular and acellular components, such as fibroblasts, myofibroblasts, pancreatic stellate cells, immune cells, blood vessels, extracellular matrix and soluble proteins such as cytokines and growth factors. The complexity of this system is explained by the concept that TME is not a static entity, but is constantly changing in composition during all the evolution phases of the PDAC, from precancer lesions to metastatic disease. When evaluating resistance to chemotherapy (*i.e.*, gemcitabine) in genetically engineered mouse models for pancreatic cancer, authors analyzed new theories and approaches to understand the importance of TME in disease pathogenesis and therapeutic response [170,171]. For example, a single point mutation in the *KRAS* oncogene was highlighted in over 90% of human PDA specimens [61], sufficient to initiate the formation of premalignant ductal transformation, the previously described PanIN. Hingorani *et al*. showed that the loss or mutation of tumor suppressor genes commonly acquired during human disease progression (TP53 and Ink4a/Arf) cooperate with Kras in mice to promote invasive cancer [172].

One of the crucial components of peritumoral stroma involved in cancer evolution is the stellate cell. Pancreatic stellate cells (PaSCs) are a rare stromal cell type normally present in the healthy pancreas [173]. Normally PaSCs are quiescent and their physiological role has yet to be delineated. Acute and chronic inflammatory conditions cause activation of PaSCs, with morphological changes, increased proliferation and expression of inflammatory compounds, such as alpha-smooth muscle actin (α-SMA) [174]. Activated PaSC are detected in areas with high collagen content. For this reason, PaSC could be involved in the pathogenesis of pancreatic fibrosis [174]. PaSCs present a limited life span in culture and for this reason a new generation of immortalized PaSC lines from human, rat and mouse pancreases have been engineered. Such immortalized PaSCs have enabled the dissection of important cross-talk pathways between PaSCs and neoplastic PDA cells by co-culturing in monolayers or three-dimensional models. PaSCs represent a resource that may explore the tumor-promoting aspects of tumor fibroblasts in PDA. Erkan *et al.* demonstrated that co-cultures of PaSCs and PDA cells increase pancreatic cancer cell proliferation and migration by release of growth factors and cytokines [175]. *In vivo* studies confirmed those findings, revealing that the co-injection of pancreatic stellate cells with tumor cells in orthotopic models of PDA increases tumor size and causes a higher incidence of metastasis [176]. A subsequent study of Xu *et al*. found that stellate cells warrant metastatic dissemination by co-migrating with neoplastic cells to potentially establish the appropriate metastatic niche or “soil” [177]. More recent publications demonstrated that PaSC *in vitro* increase the stem cell phenotype of pancreatic cancer cells, suggesting for the first time a possible pharmacological target with potential additional benefits [178]. The signalling pathways activated in PaSCs in response to contact with cancer cells will be an interesting platform on which to develop therapies targeting PaSCs, *i.e.*, MAP kinase, PDGF, FGF, transforming growth factor β (TGFβ), connective tissue growth factor (CTGF) and epidermal growth factor (EGF) [179].

In PDAC histology we can observe an abundance of extracellular matrix (ECM), commonly referred to as desmoplasia. The accumulation of ECM components (collagen, fibronectin, proteoglycans and hyaluronic acid) distorts the normal architecture of pancreatic tissue inducing an abnormal configuration of blood and lymphatic vessels [180]. The rigidity of the ECM that compresses blood vessels is able to reduce perfusion and delivery of drugs to neoplastic cells, thus contributing to therapeutic resistance in PDA [181]. Sonic hedgehog (SHH) signaling has been shown to be restricted to the stromal compartment and enhance the desmoplastic reaction [182], so pharmacological inhibition of the SHH pathway may positively impact on drug (*i.e.*, gemcitabine) delivery. Several clinical trials have been initiated as a result of this and are recruiting patients to investigate the mechanism and treatment effect of pharmacological SHH-inhibitors in pancreatic cancer patients [183–186].

Secreted protein acidic and rich in cysteine (SPARC) represents another proposed target to facilitate depletion of the tumor stroma in pancreatic cancer. SPARC is overexpressed by fibroblasts in the TME of human and murine PDA and has been shown to inversely correlate with survival [187]. On this basis, a novel drug formulation consisting of paclitaxel associated with albumin (Abraxane or nab-paclitaxel) has been hypothesized to accumulate in and potentially deplete PDA tumor stroma via binding of albumin to SPARC-positive fibroblasts, thus representing a mechanism for targeting a specific cell type within the PDA tumor microenvironment [188]. The first clinical trial of gemcitabine in combination with nab-paclitaxel showed a promising overall survival with elevated SPARC expression correlated with increased survival. Patients with high SPARC levels had a mean overall survival of 17.8 months as compared to 8.1 for low SPARC [189]. Further in-depth investigations are necessary to elucidate the exact role of SPARC as a novel biomarker for PDA patients.

Similar to other cancer types, inflammation also seems to be crucially linked to PDA development, exemplified by the fact that chronic pancreatitis is a major risk factor [190]. Immunosuppressive cell types such as regulatory T cells and myeloid derived suppressor cells (MDSCs) are predominant with hardly any cytotoxic T lymphocytes (CTLs) infiltrating the tumor. Successful immunotherapy depends on the cancer cells expressing proteins that can be recognized as altered by the immune system (*i.e.*, Tumor Specific Antigens, TSA). The goal of these studies is to induce high-affinity cytotoxic T cells (CTL or CD8 T cells) against tumor cells without causing autoimmunity. Antigens targeted in immunotherapy clinical trials in PDA have included Muc1, mesothelin, Kras, carcinoembryonic antigen (CEA), survivin and telomerase. Several trials are warranted in future experimental analysis [191].

In summary, growing evidence suggests that extensive desmoplastic reaction may be at least responsible for the innate chemoresistance in pancreatic tumors, and therapeutic benefit may be gained by strategies aimed at depleting the desmoplastic stroma, ally of cancer cells against chemotherapeutic drugs.

## 7. Conclusions and Future Perspectives

Intensive research over the last few years has shown that pancreatic cancer is fundamentally a chameleonic and dynamic disease, with determinant etiological aspects, such as genetic factors, correlations between cancer cells and peritumoral stroma, and immune system activity. The genetic disease determines inherited germline and/or acquired somatic mutations in cancer-associated genes. For this reason, it has uncovered multiple alterations in many genes that are critical in PDAC progression. Treating PDAC by targeting the tumour microenvironment is another strategy, as reported, for many aspects: the promotion of tumour-eliminating processes, the suppression of tumour-promoting inflammation, and the modulation of the protective fibrotic stroma of PDAC to allow access to tumour epithelium by conventional chemotherapeutics. Only the accurate study and knowledge of the complex microenvironment can provide an excellent tool linking basic science with clinical application. The potential for preoperative characterization of PDAC and the direction of tumour-specific individualized therapy is within sight. Individualised multi-targeted therapy is likely to be necessary in order to treat PDAC effectively, as high recurrence rates following surgery and late presentation of disease remain significant hurdles. Inflammation and peritumoral stroma are implicated in the earliest stages of PDAC tumorigenesis, in early metastases, and in tumour progression. Combinatorial therapeutic regimens must look to capitalise on the importance of this tumour-microenvironment relationship. As seen in this review, targeting EMT could also contribute to increased sensitivity to standard chemotherapy and to promising growth factor directed therapies, such as those against EGFR signaling. By attenuating fibrosis, it can also increase delivery of drugs to cancer cells. Targeting EMT can also reduce the population of cancer stem cells that are thought to contribute to metastatic disease and treatment resistance.

In addition, an increased understanding of the molecular basis of the disease has provided the identification of new drug targets enabling rational drug design, and facilitated the production of animal and *in vitro* models of the disease on which such therapies can be tested. The poor prognosis and late presentation of pancreatic cancer patients emphasize the importance of early detection, which will lead to future clinical trials in the fight against pancreatic cancer. In this context, a rapid discovery of effective biomarkers remains crucial. Knowledge of tests recognizing genetic alterations and molecular events typical of PDAC represents the first step to a new therapeutic approach for this still aggressive neoplasm.

## Figures and Tables

**Table 1:** Oncogenes in PDAC: genetic aberration percentual and type.

Molecular target	Genetic aberration (%)	Type aberration
*EGFR*	43%–69%	overexpression
*VEGF*	93%	overexpression
*KRAS*	90%	point mutation
*BRAF*	30%	point mutation
*AKT2*	15%	amplification
*MYB*	10%	amplification
*IGF-1R*	64%	amplification
*MMPs*	85%	amplification
*Hedgeogh*	70%	amplification
*m-TOR*	65%	overexpression
*MEK*	72%	point mutation
*COX-2*	67%–90%	point mutation

**Table 2:** Tumor suppressors in PDAC: genetic aberration percentage and type.

Molecular target	Genetic aberration (%)	Type aberration
*p16INK4A/CDKN2A*	40%	deletion
*P53*	50%–75%	intragenic mutation
*DPC4* (*Smad4*)	30%	deletion
